# Supplementary material for: Utilizing the scale-invariant feature transform algorithm to align distance matrices facilitates systematic protein structure comparison
Source: Bioinformatics. 2024 Feb 6;40(3):btae064. doi: 10.1093/bioinformatics/btae064 (PMC10924749; doi:10.1093/bioinformatics/btae064)
Supplement: btae064_Supplementary_Data [file btae064_supplementary_data.zip › Supplementary materials.pdf]

Figure S1

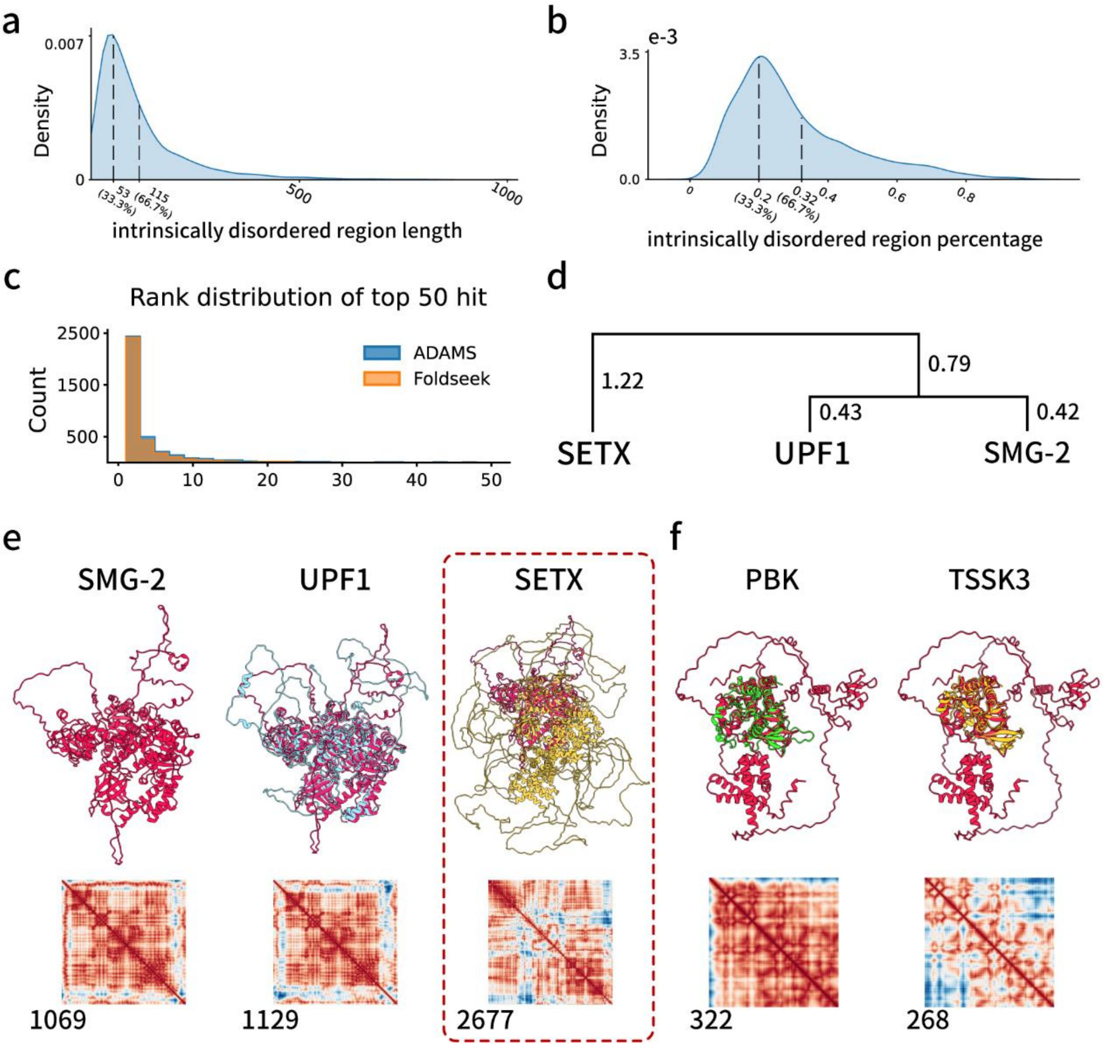

Figure S1. ADAMS filter noise from IDR rich proteins

a) Length distribution of intrinsically disordered regions in human proteins.

b) Percentage of intrinsically disordered regions in human proteins.

c) Distribution of positive hit rank of the positive cases when the positive case was in top 50 hit of both methods.

d) A phylogenetic tree illustrates the relationship between proteins identified by ADAMS and Foldseek during the search for SMG-2.

e) The results of searching for SMG-2 in the human protein database are provided by both Foldseek and ADAMS. ADAMS successfully identified UPF1(1). Foldseek erroneously labeled a structure as a top-ranked match, highlighted with red dashed lines, which was later confirmed to be a false positive result.

Figure S2

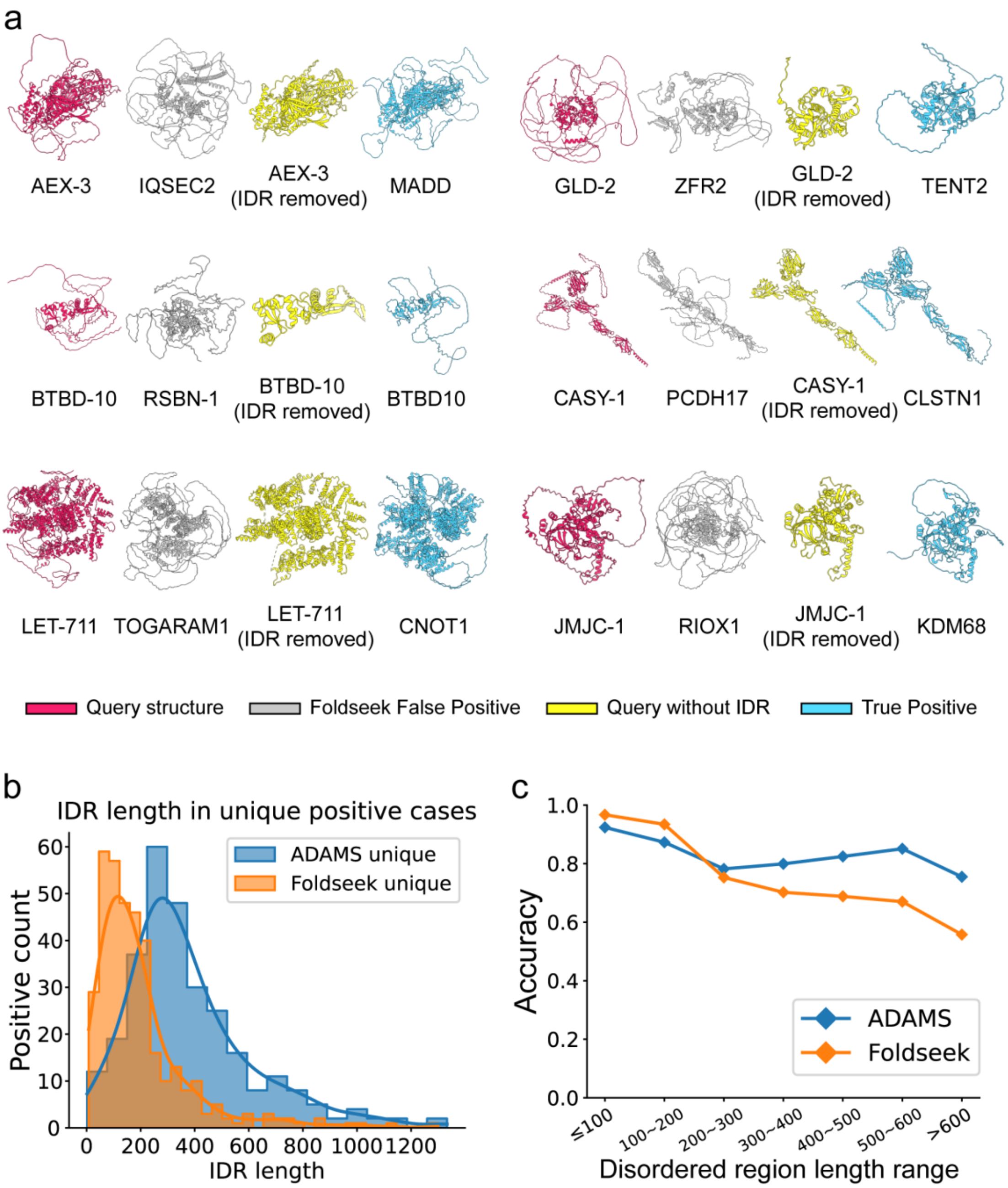

Figure S2. Robust Structure Comparison by ADAMS in the Presence of IDR Noise.

a) The figure showcases six examples wherein Foldseek initially produced false positive results. However, upon removal of IDR regions, these cases successfully identified the correct homolog. The red structures represent the original protein structures, while the yellow structures depict the same structures with IDR regions removed. The initial top hit, originally a false positive (colored in gray), and the corrected homolog (colored in blue) are highlighted. This demonstrates instances where Foldseek's false positive cases were rectified by removing IDR regions during the search process. b) IDR length distribution in unique positive cases of Foldseek and ADAMS on the

OrthoList2 benchmark. c) Correct rate of ADAMS and Foldseek on protein homolog searching with different range of disordered region length on the OrthoList2 benchmark.

Query	Foldseek False Positive	True Positive
GLD-2	ZFR2	TENT2
IPLA-1	SEC23IP	DDHD1
BTBD-10	RSBN1	BTBD10
NRDE-2	SF3B1	NRDE2
AEX-3	IQSEC	MADD
CASY-1	PCDH17	CLSTN1
DSH-1	TIAM2	DVL1P1
EGL-8	MPRIP	PLCB1
CDK-8	CDKL5	CDK8
LIN-35	PKP1	RB1
LIN-45	DDR2	BRAF
LIN-49	JADE1	BRPF1
MYRF-2	CAMTA2	MYRFL
PAR-1	NIM1K	MARK1
RAN-3	RPGR	RCC1
SMG-2	SETX	UPF1
TAF-1	TAF1L	KIF14
RAN-3	RPGR	RCC1
KLP-7	KIF14	KIF2A
LIN-41	TENM3	TRIM71

Table S1. IDR noise removal improved the search performance of Foldseek.
