# Supplementary figures and images for: Utilizing the scale-invariant feature transform algorithm to align distance matrices facilitates systematic protein structure comparison
Source: Bioinformatics. 2024 Feb 6;40(3):btae064. doi: 10.1093/bioinformatics/btae064 (PMC10924749; doi:10.1093/bioinformatics/btae064)

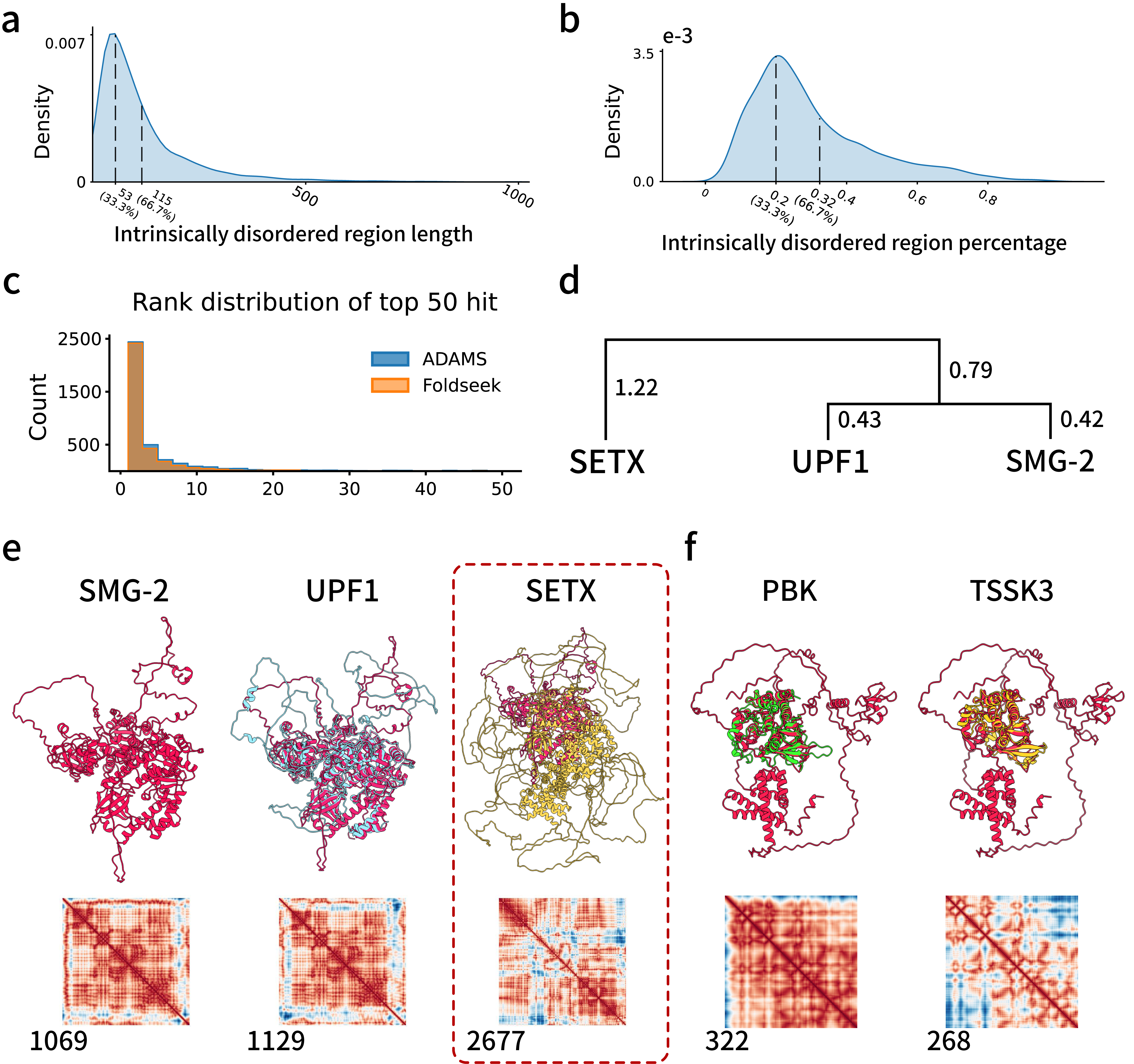

Supplement: btae064_Supplementary_Data [file btae064_supplementary_data.zip › FigureS1.tif]
